# A microRNA diagnostic biomarker for amyotrophic lateral sclerosis

**DOI:** 10.1093/braincomms/fcae268

**Published:** 2024-09-13

**Authors:** Sandra Anne Banack, Rachael A Dunlop, Paul Mehta, Hiroshi Mitsumoto, Stewart P Wood, Moon Han, Paul Alan Cox

**Affiliations:** Brain Chemistry Labs, Jackson, WY 83001, USA; Brain Chemistry Labs, Jackson, WY 83001, USA; Office of Innovation and Analytics, Agency for Toxic Substances and Disease Registry, Centers for Disease Control and Prevention, Atlanta, GA 30033, USA; Eleanor and Lou Gehrig MND/ALS Research Center, Department of Neurology, Columbia University Medical Center, New York, NY 10032, USA; Brain Chemistry Labs, Jackson, WY 83001, USA; Office of Innovation and Analytics, Agency for Toxic Substances and Disease Registry, Centers for Disease Control and Prevention, Atlanta, GA 30033, USA; Brain Chemistry Labs, Jackson, WY 83001, USA

**Keywords:** reproducibility, MND, sensitivity, AUC, predictive value

## Abstract

Blood-based diagnostic biomarkers for amyotrophic lateral sclerosis will improve patient outcomes and positively impact novel drug development. Critical to the development of such biomarkers is robust method validation, optimization and replication with adequate sample sizes and neurological disease comparative blood samples. We sought to test an amyotrophic lateral sclerosis biomarker derived from diverse samples to determine if it is disease specific. Extracellular vesicles were extracted from blood plasma obtained from individuals diagnosed with amyotrophic lateral sclerosis, primary lateral sclerosis, Parkinson’s disease and healthy controls. Immunoaffinity purification was used to create a neural-enriched extracellular vesicle fraction. MicroRNAs were measured across sample cohorts using real-time polymerase chain reaction. A Kruskal–Wallis test was used to assess differences in plasma microRNAs followed by *post hoc* Mann–Whitney tests to compare disease groups. Diagnostic accuracy was determined using a machine learning algorithm and a logistic regression model. We identified an eight-microRNA diagnostic signature for blood samples from amyotrophic lateral sclerosis patients with high sensitivity and specificity and an area under the curve calculation of 98% with clear statistical separation from neurological controls. The eight identified microRNAs represent disease-related biological processes consistent with amyotrophic lateral sclerosis. The direction and magnitude of gene fold regulation are consistent across four separate patient cohorts with real-time polymerase chain reaction analyses conducted in two laboratories from diverse samples and sample collection procedures. We propose that this diagnostic signature could be an aid to neurologists to supplement current clinical metrics used to diagnose amyotrophic lateral sclerosis.

## Teaser

Banack *et al*. report that a blood-based, eight-microRNA fingerprint is diagnostic for amyotrophic lateral sclerosis. Gene fold regulation was consistent across four separate patient cohorts. Diagnostic accuracy indicated high sensitivity and specificity, an area under the curve of 98% and clear statistical separation from neurological controls.

## Graphical Abstract

**Graphical Abstract:**
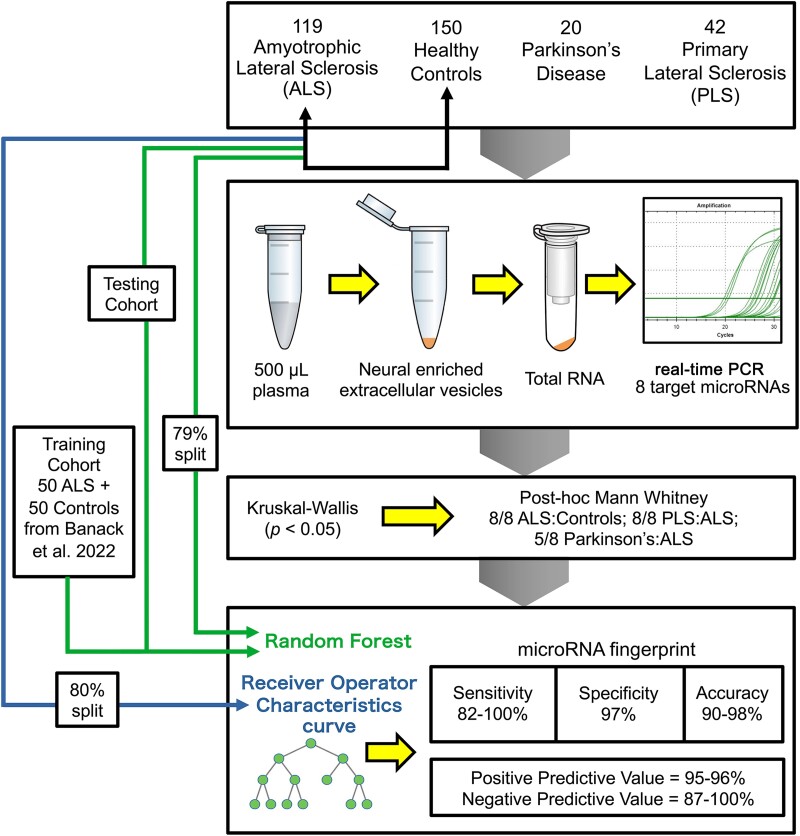

## Introduction

The need for biomarkers for all neurodegenerative diseases is well established.^1,2^ Biomarkers are essential for reducing diagnostic delays and improving disease outcomes as well as for therapeutic drug development. To improve reproducibility and ultimately to achieve biomarker adoption, every biomarker should undergo robust validation.^3^ The most effective biomarkers are reliable measures of disease (or disease state), applicable across drug development trials and independent of the drug being tested.

Amyotrophic lateral sclerosis is a rare neurological disease with an estimated 30 000 active cases per year in the USA. Incident rates in 2018 were 1.6 per 100 000 US population, and prevalence was 9.1 per 100 000 population.^4^ The disease typically develops during mid-to-late life, progresses rapidly and is terminal within 2–5 years, but some patients survive much longer.^4-6^ The clinical presentation of amyotrophic lateral sclerosis is also variable with distal muscle limb weakness more common than bulbar onset.^7^ Familial amyotrophic lateral sclerosis represents approximately 10% of all cases with the remaining 90% of sporadic cases occurring in individuals with no known genetic mutations.^8^ Care for amyotrophic lateral sclerosis patients remains supportive and palliative, while a cure, and even meaningfully effective therapy, remains elusive.^7^

An amyotrophic lateral sclerosis diagnosis is made by a clinician after observation of progressive degeneration of upper and lower motor neurons using standardized clinical criteria.^5,6,8^ However, patients frequently experience diagnostic uncertainty resulting in delayed diagnosis, an increased number of physician consultations and sometimes unnecessary medical procedures.^9^ Misdiagnosis can be as high as 68%^9^ partly because family physicians and some general neurologists outside of large metropolitan areas do not observe many cases in their lifetimes. A robust amyotrophic lateral sclerosis diagnostic biomarker would give patients a chance at earlier treatment and novel therapeutic intervention. It could also accelerate the testing of new drug candidates in clinical trials.^10^

Amyotrophic lateral sclerosis biomarker research, both diagnostic^11^ and prognostic,^12^ is ongoing, with recent developments in neuroimaging, electrophysiology and fluid-based markers.^13^ Imaging biomarkers can be difficult for amyotrophic lateral sclerosis patients as symptom-related complications make these procedures uncomfortable.^14^ Blood or urine-based biomarkers are generally considered preferable to cerebrospinal fluid markers because they are less invasive. Two candidates, neurofilament and p75 neurotrophin receptor, are being investigated as pharmacological markers in conjunction with amyotrophic lateral sclerosis clinical trials.^14-16^ RNA biomarkers are also of interest as amyotrophic lateral sclerosis biomarkers (e.g. Joilin *et al*.,^17^ Zhu *et al*.,^18^ Shen *et al*.^19^ and Grima et al.^20^) and, in combination with neurofilament, may have promise in survival prognostication.^12^ A recent study has demonstrated the potential of HDGFL2, a cryptic neoepitope, as a marker of pre-symptomatic amyotrophic lateral sclerosis.^21^

Our objective was to develop a blood-based diagnostic amyotrophic lateral sclerosis biomarker which could reliably identify patients who had previously been diagnosed using standardized clinical criteria. We hypothesized that patterns and concentrations of microRNAs (miRNA) present within neural-enriched extracellular vesicles can be used with sensitivity and specificity to aid an amyotrophic lateral sclerosis diagnosis.

We report an amyotrophic lateral sclerosis diagnostic eight-miRNA fingerprint (miR-199a-3p, miR-4454, miR-10b-5p, miR-151a-5p, miR-199a-5p, miR-151a-3p, miR-146a-5p and miR-29b-3p) derived from neural-enriched extracellular vesicles obtained from a standard clinical blood draw. miRNAs were identified from an earlier experiment on blood plasma from neural-enriched extracellular vesicles in which 101 miRNAs were shown by next-generation sequencing to be differentially expressed between amyotrophic lateral sclerosis patients and healthy controls.^22^ Thirty-four miRNAs were chosen, due to their magnitude of dysregulation, for validation using real-time polymerase chain reaction (PCR).^22^ The 8 miRNAs used in this study were chosen from 34 miRNAs because they consistently and statistically separated amyotrophic lateral sclerosis patients and control patients following quantitative PCR (qPCR) experiments on 3 different cohorts of patient and control samples.^23^ The bilipid membrane of the extracellular vesicles contributes to reproducibility in two ways: (i) protecting miRNA from degradation and (ii) facilitating purification of the sample by immunopurification through transmembrane proteins on the surface of the extracellular vesicles.^23,24^ This miRNA fingerprint fills an unmet amyotrophic lateral sclerosis drug development and medical diagnostic need and could facilitate the identification of amyotrophic lateral sclerosis at its earliest stages, thereby reducing diagnostic uncertainty. Here, we present data from a large fourth cohort of patients including amyotrophic lateral sclerosis, neurological controls with a diagnosis of Parkinson’s disease, neurological controls with a diagnosis of primary lateral sclerosis and healthy controls with no known neurological symptoms to test the hypothesis that this eight-miRNA fingerprint could be used, along with current diagnostic tools, to aid clinical amyotrophic lateral sclerosis diagnosis.

## Materials and methods

### Study design

Using retrospective blood samples from previously identified amyotrophic lateral sclerosis, primary lateral sclerosis, Parkinson’s disease patients and healthy controls, we evaluated an eight-miRNA fingerprint as an aid to diagnosing amyotrophic lateral sclerosis.

### Blood plasma

Blood plasma samples from 119 amyotrophic lateral sclerosis, 42 primary lateral sclerosis, 20 Parkinson’s disease patients and 150 healthy controls were evaluated for miRNAs contained within neural-enriched extracellular vesicles (see Table 1 for the study cohort characteristics). Primary lateral sclerosis is considered an amyotrophic lateral sclerosis mimic, and Parkinson’s disease was also included for comparison as a neurological control.^25^ Sample number was limited by the availability of samples. Sample inclusion eligibility was based on diagnosis for amyotrophic lateral sclerosis, primary lateral sclerosis or Parkinson’s disease and the absence of a neurological diagnosis for healthy control samples. Subjects’ consent was obtained according to the Declaration of Helsinki and was approved by the ethical committee of each institution.

**Table 1:** Characteristics of study cohort

	Amyotrophic lateral sclerosis	Healthy control	Parkinson’s disease	Primary lateral sclerosis	Data used for machine learning training data set^23^	
					Amyotrophic lateral sclerosis	Healthy control
Total samples	119	150	20	42	50	50
Male/female	72/28	110/40	14/6	24/17	35/15	30/20
Age group						
<30	0	5	0	0	2	14
30–39	3	17	0	1		13
40–49	14	15	0	5	7	9
50–59	21	86	5	12	19	10
60–69	32	26	3	16	15	4
70–79	28	1	9	4	6	0
80–89	2	0	3	3	1	0
ALSFRS-R						
Mean, median (min–max)	32, 33.5 (6–45)					
Early stage ≥ 35 ALSFRS	50					
Late stage < 35 ALSFRS	50					
Progression						
ALSFRS-R slope < −0.5	9				8	
ALSFRS-R slope −0.5 to −1.0	9				10	
ALSFRS-R slope > −1.0	0				6	
Onset						
Speech and/or swallowing muscles	8				5	
Arm or hand	25				8	
Neck, back or abdominal area	7				0	
Leg or foot	35				8	
Breathing muscles	2					
All over body	2					
Amyotrophic lateral sclerosis recognized in other family members	3				0	
Months since diagnosis						
Mean, median (min–max)	16.5, 6.5 (2–122)		59, 54.5 (2–178)	68.1, 51.9 (9.3–172.9) ^a^		
Other						
Idiopathic Parkinson’s disease			13			
Parkinson-plus syndromes			7			

Primary lateral sclerosis samples from the natural history study.^26^ Amyotrophic lateral sclerosis progression data were calculated on all samples wherein at least two data points were provided in the accompanying database. Complete individual characteristics for all patients were not available in the respective databases.

^a^Disease duration at baseline.

We accessed 100 amyotrophic lateral sclerosis blood samples, collected across the USA between 2017 and 2018, through the US National ALS Biorepository, maintained by the Centers for Disease Control and Prevention, and the Agency for Toxic Substances and Disease Registry [CDC, Advarra Institutional Review Board (IRB) Pro00053269]. Nineteen amyotrophic lateral sclerosis blood samples came from a Phase IIa clinical trial (NCT03580616, Dartmouth IRB D18095, collected between 2019 and 2022). We used 42 blood plasma samples from the multi-site primary lateral sclerosis natural history study by Dr Hiroshi Mitsumoto (approved by the Columbia University IRB and by the IRB at each individual site; samples were collected in 2021–23 following the protocols set forth in Mitsumoto *et al*.^26^). Primary lateral sclerosis is characterized clinically by pure upper motor neuron dysfunction; however, the time since symptom onset is widely accepted by investigators as part of the diagnostic criteria. Patients used in this study fell into three categories: (i) ‘early primary lateral sclerosis’ we arbitrarily defined as having upper motor neuron signs present for less than 2 years after symptom onset (*n* = 4); (ii) ‘probable primary lateral sclerosis’ with symptom onset between 2 and 4 years (*n* = 18); and (iii) patients with a disease duration longer than 4 years since symptom onset, which is ‘definite primary lateral sclerosis’ (*n* = 19). However, no patients were admitted with a disease duration more than 15 years. We obtained 20 Parkinson’s disease blood plasma samples from Precision for Medicine (Bethesda, MD), collected across the USA between 2018 and 2020 (Norton, MA, CR00425931). The 150 controls, without known neurological disease, were sourced from Innovative Research Inc. [Novi, MI, Food and Drug Administration (FDA) approval, #3003372368, collected across the USA prior to 2022] and Precision for Medicine (Norton, MA, CR00425931, collected across the USA from 2020 to 2022) and were considered to be healthy controls. Selection of these healthy controls was chosen to match the gender and age characteristics of the CDC ALS cohort where possible. Informed consent from all participants was obtained through the respective institutions involved. Since this study used only de-identified participant data, Advarra IRB (Pro00053269) determined that it ‘*does not meet the DHHS definition of human subjects research under 45 CFR 46 and, therefore, does not require IRB oversight*”. During the laboratory analyses, researchers were blinded as to the status of the samples and whether they represented disease or control patients.

Blood samples were collected in K_2_EDTA tubes, and because the samples were obtained from different institutions, plasma processing protocols varied. Samples were blinded before being processed and analysed.

### Extracellular vesicle isolation

Extracellular vesicles (EVs) were processed as reported in our prior studies using polymer-based precipitation and immunoassay purification.^22,23^ Characterization of EVs and differences between total EV collection, neural-enriched EV, and the total minus neural-enriched extracellular vesicles fraction has been previously published.^22,24^

### RNA extraction

Previous studies have described extraction of total RNA retaining short RNA species.^27^ We conducted extraction according to the instructions in Qiagen RNeasy Midi Kit Part 2: RNA isolation. We added spike-ins for monitoring RNA extraction efficiency (UniSp2, UniSp4 and UniSp5), available as part of the RNA Spike-in Kit, for RT (Qiagen, cat. no. 339390), at 1 µL per 700 µL lysis buffer (see Dunlop *et al*.,^27^ for a detailed modified protocol).

### cDNA synthesis

We synthesized cDNA using the miRCURY LNA RT Kit (Qiagen, cat. no. 339340) according to the manufacturer’s instructions (see Supplementary Table 1 for reaction conditions) and as described previously.^27^ Modifications included the addition of 1 µL of a spike-in control mix containing UniSp6 and *Caenorhabditis elegans cel*-miR-39–3p to each cDNA reaction to monitor cDNA synthesis [reverse transcription (RT)] efficiency. UniSp6 is provided in the miRCURY LNA RT kit, and *cel*-miR-39–3p is included in the RNA Spike-in Kit, for RT. We used 4 µL of total RNA rather than 2 µL as recommended by the manufacturer, since we have previously determined that 4 µL returns a more robust signal.^27^ We conducted each 10 µL cDNA synthesis reaction in duplicate and pooled them to create a total of 20 µL cDNA. Prior to storage at −20°C, we aliquoted cDNA into 3 µL aliquots to avoid multiple freeze/thaw cycles.

### Real-time quantitative PCR quality control

Following cDNA synthesis, we ran quality control (QC) qPCR for every sample on eleven QC miRNA targets. To determine RNA extraction efficiency, we measured Cqs for the spike-ins UniSp2, UniSp4 and UniSp5. To determine if RT (cDNA synthesis) proceeded without inhibition, we measured the spike-ins UniSp6 and cel-miR-39–3p. Sample signal is a measure of the amount of cDNA in each sample and determines whether samples are suitable for downstream analysis. For determination of sample signal, we measured six endogenous miRNAs: miR-142–3p, miR-451a, miR-23a-3p, miR-30c-5p, miR-103a-3p and miR-191-5p.^28^

### qPCR of miRNA using SYBR green

We conducted qPCR for all QC, target and reference miRNA using Qiagen miRCURY LNA miRNA SYBR PCR Assays (cat. no. 339306; Supplementary Table 2 for GeneGlobe IDs) and the miRCURY LNA™ SYBR Green PCR Kit (Qiagen, cat. no. 339347) according to the manufacturer’s instructions (Supplementary Table 3) and as described previously.^27^ Briefly, we diluted cDNA 1/30 into nuclease-free water and used 3 µL in each 10 µL qPCR reaction. We automated the pipetting of master mix (7 µL) and samples (3 µL) into 384-well plates using the Opentrons OT-2 liquid-handling robot using a custom protocol written in Python. qPCR was conducted on the Bio-Rad CFX Opus 384 in 384-well plates (Bio-Rad Hard Shell PCR Plates, thin-wall, cat. no. HSP3805). We acquired data in Bio-Rad CFX Maestro version 2.3 after 40 cycles. Each plate contained triplicate inter-plate calibrators (TATAA IPC, TATAA Biocenter, cat. no. IPC250S) pipetted into the same wells on every plate, to enable comparison of samples run in January 2023 with samples run in August 2023. We applied an IPC correction factor to all samples to enable cross-plate comparison. We included a melt curve and triplicate no-template controls in each assay to check for primer specificity and any non-specific amplification.

### Haemolysis calculation

Samples that have undergone haemolysis may show artificially increased miRNA signals, which could distort subsequent results. The gold standard method for assessing haemolysis is considered a comparison of two miRNA, where miR-23a-3p is stable and not affected by haemolysis while the expression of miR-451a is erythrocyte specific with increased concentrations when haemolysis is present.^29^ We, therefore, calculated a delta Cq (ΔCq) for miR-23a-3p minus miR-451a where larger values indicate potential haemolysis.^30^ We flagged ΔCq ≥ 7 for further in-depth evaluation according to standard protocols.^29^ The highest result was 8.66 (S141 from January 2023) with a handful of samples returning values ≥ 7 ≤ 8.30. The miRNA Cq values for these samples were otherwise within acceptable ranges as determined using the interquartile range (Q1 = 30.6, Q3 = 34.9, IQR = 4.3, interquartile multiplier value *k* = 1.5), so we did not exclude them from further analysis (see Supplementary Tables 4 and 5 for haemolysis results).

All samples passed QC and were deemed suitable for downstream analysis.

### Relative quantitation using 2^−(ΔΔCq)^

A NormFinder analysis confirmed the stability of miRNAs used for normalization, and the same miRNAs as those used in prior experiments were chosen for consistency and reproducibility (miR-146a-5p, miR-29b-3p and miR-126-5p).^22,23^ We used the geometric mean for relative quantitation/normalization and performed a suitability check following Vandesompele *et al*.^31^ Since the standard deviation of the ratio V ¾ was less than 0.15, no additional reference miRNAs were needed.^31^ We calculated gene fold expression changes using 2^−(ΔΔCq)^, with ΔΔCq calculated as the normalized sample Cq value minus the mean of the normalized control sample Cqs. We calculated fold regulation as relative mean gene fold expression changes in amyotrophic lateral sclerosis /controls, Parkinson’s disease/amyotrophic lateral sclerosis and primary lateral sclerosis/amyotrophic lateral sclerosis, respectively, and define fold regulation as equal to gene fold change when greater than one and as negative one divided by gene fold change when fold change was less than one.

### Statistical analyses

The skewed distribution of the data which varied between miRNA suggested that a non-parametric statistical analysis would be preferable. We performed a Kruskal–Wallis test to assess two alternative hypotheses at the level of *P <* 0.05:

H_0_: The gene fold changes, 2^−(ΔΔCq)^, of miRNA from all disease groups were the same.

H_1_: The gene fold changes, 2^−(ΔΔCq)^, of miRNA from all disease groups were not the same.

If the null hypothesis H_0_ was to be rejected, we would perform *post hoc* analyses on gene fold change values (2^−(ΔΔCq)^) between the following pairs of disease groups, amyotrophic lateral sclerosis versus controls, amyotrophic lateral sclerosis versus primary lateral sclerosis and amyotrophic lateral sclerosis versus Parkinson’s disease, using Mann–Whitney tests, at the level of *P* < 0.05 to test the following alternative hypotheses:

H_0_: There is no difference in the median gene fold change, 2^−(ΔΔCq)^, for miRNA between the two disease groups.

H_1_: There is a difference in the median gene fold change, 2^−(ΔΔCq)^, for miRNA between the two disease groups.

Individual miRNAs (2^−(ΔΔCq)^) representing values greater than four standard deviations from the mean were identified as extreme outliers and removed before gene fold expression calculation (one from Parkinson’s disease and five from control samples). Three samples were considered invalid and removed entirely from gene fold expression calculations (one amyotrophic lateral sclerosis, one control and one primary lateral sclerosis) because ≥50% of the miRNA values exceeded four standard deviations from the mean suggesting that the plasma samples from these individuals were compromised. Although nothing was flagged for these samples in the quality control measures built into our methods, we note that plasma samples were collected, processed and stored initially by numerous individuals across the USA. Finding three samples of concern out of 331 total represents less than 1% of the total samples analysed.

We evaluated sensitivity and specificity using normalized ΔCq values. Sensitivity is a measure of the ability of a screening test to positively classify someone who has the disease. It is calculated as a per cent identification of true positive cases in the data set divided by the total number of individuals with the disease, as defined by a gold standard method of disease identification.^32^ The current gold standard for an amyotrophic lateral sclerosis diagnosis is a clinical evaluation by a neurologist who has evidence of progressive upper and lower motor neuron degeneration as seen in EMG, the ALSFRS-R score, and forced vital capacity based upon ratified consensus criteria.^7^ Specificity is a measure of the ability of a screening test to accurately classify someone who does not have the disease, comparing true negative cases to the total number of individuals without the disease in the data set. Also important for biomarker evaluation is a measure of positive predictive value (PPV) for the test, which compares true positive cases with false positive identification. The negative predictive value (NPV) of a test is the number of true negative cases identified by the test relative to false negative identification.^32^ We used three orthogonal measures to determine amyotrophic lateral sclerosis classification accuracy against healthy controls in RStudio.^33-37^ (i) A logistic regression was calculated to determine a receiver operator characteristics (ROC) curve using only the current data and a random 80% split for training and 20% for testing purposes. Values from all amyotrophic lateral sclerosis and non-neurological controls (*n* = 269) were included in the model without removing outliers. Seed set was 375, and all miRNA data were included in this model. (ii) A random forest machine learning algorithm was used applying all of the current data from this paper (*n* = 269) without removing outliers and a 79% split between training and testing data. The model was tested with a seed set of 375 and iteratively removing miRNA with low model contribution. (iii) A second random forest algorithm was used testing the current data set (*n* = 269) against a training set comprised of ΔCq values from an independent cohort previously published (*n* = 100, Banack *et al*.^22^; Table 1). The model was tested with a seed set of 101 and by iteratively removing miRNA with low contributions to the classification. Parkinson’s disease and primary lateral sclerosis samples were not included in these models due to sample sizes that were too low to give meaningful results.^38^

## Results

**Figure 1:**
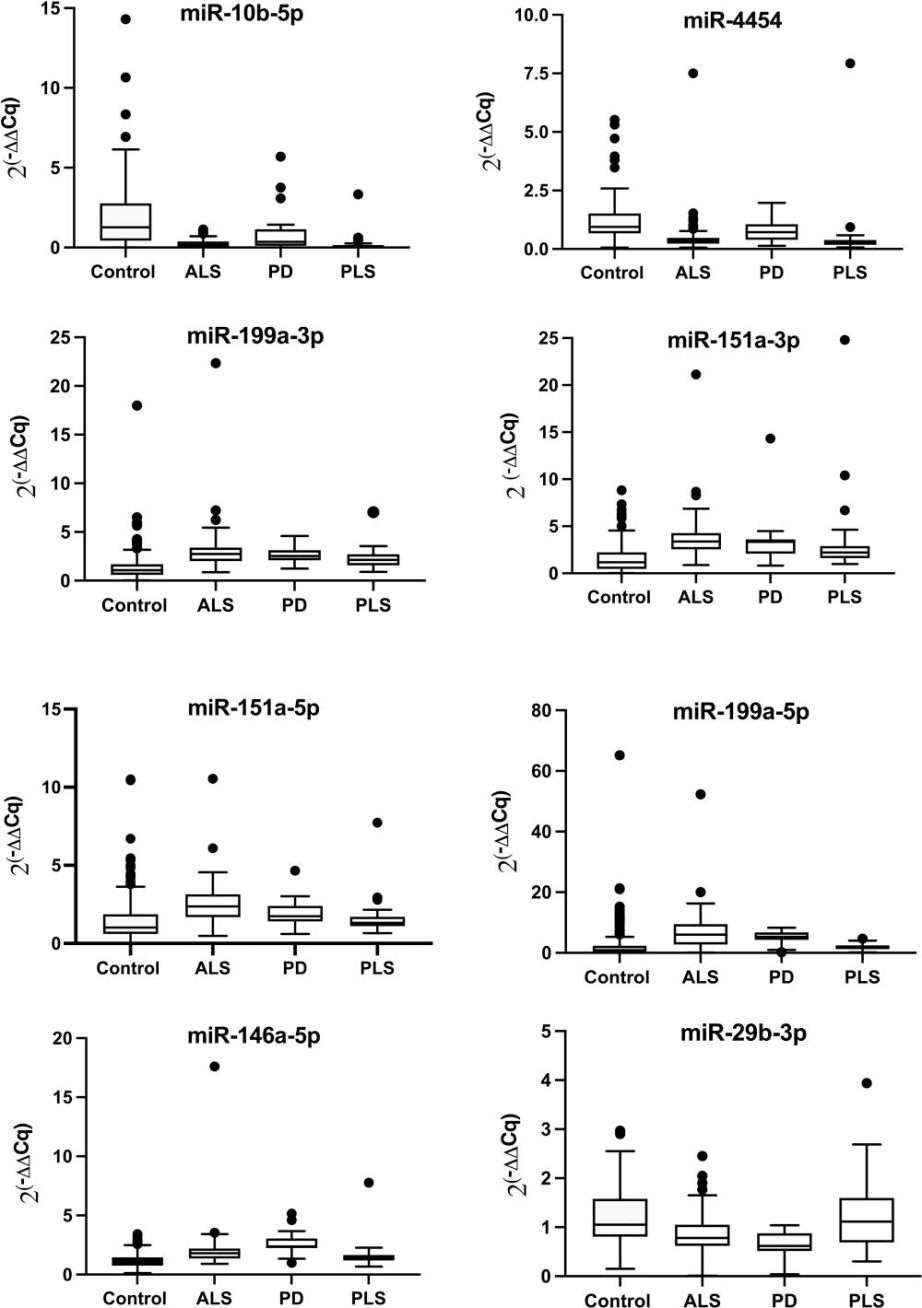
**Gene fold expression.** Variability in gene fold expression (2^(−ΔΔCq)^, *x*-axis) between amyotrophic lateral sclerosis (*n = 119*), Parkinson’s disease (*n = 20*), primary lateral sclerosis (*n = 42*) and control samples (*n = 150*) represented by boxplot for eight miRNAs. Center lines show the medians; box limits indicate the 25th and 75th percentiles; whiskers extend 1.5 times the interquartile range from the 25th to 75th percentiles; outliers are represented by circles. Two extreme values (282 and 275) from control samples were removed from miR-4454 to best illustrate the data (see Table 3 for the statistical analyses).

A comparison of the eight-miRNA amyotrophic lateral sclerosis fingerprint between 119 amyotrophic lateral sclerosis and 150 healthy controls indicated identical fold regulation direction with similar magnitude to those found in prior published studies (Table 2).^22,23^ A comparison of median gene fold change between amyotrophic lateral sclerosis, Parkinson’s disease, primary lateral sclerosis and healthy controls using a Kruskal–Wallis analysis was highly significant for all eight miRNAs with H values ranging from 38 to 136 (critical value = 16, *P* < 0.001), suggesting that at least one of the disease groups was not from the same population. Given the non-normal distribution of miRNA fold change values (Fig. 1), we used a *post hoc* Mann–Whitney analyses to compare the median gene fold change (2^−(ΔΔCq)^) for each miRNA for the following disease groups: amyotrophic lateral sclerosis versus healthy controls, Parkinson’s disease versus amyotrophic lateral sclerosis and primary lateral sclerosis versus amyotrophic lateral sclerosis. For all eight miRNAs, the gene fold change between amyotrophic lateral sclerosis and control samples had *P* < 0.05 (Table 3). The same was true between primary lateral sclerosis and amyotrophic lateral sclerosis. For the comparison between Parkinson’s disease and amyotrophic lateral sclerosis, five out of eight miRNAs were different. Interestingly, the three miRNAs that were not identified as different between ALS and Parkinson’s disease on the Mann–Whitney analysis were also not different in another study comparing amyotrophic lateral sclerosis with controls and may be an artefact of a small sample size.^23^

**Table 2:** Fold regulation of an-eight miRNA amyotrophic lateral sclerosis fingerprint across 449 independent plasma samples over four unique cohort experiments showed remarkable consistency in both direction and values

	Fold regulation			
	Current (*n* = 269)	Banack *et al*.^23^ (*n* = 100)	Banack *et al*.^22^ experiment 1 (*n* = 40)	Banack *et al*.^22^ experiment 2 (*n* = 40)
miR-10b-5p	(−7.38)	(−3.2)	(−2.1)	(−7.0)
miR-4454	(−2.55)	(−2.7)	(−1.7)	(−1.8)
miR-199a-3p	2.03	1.0	1.4	2.7
miR-151a-3p	2.09	1.1	1.5	2.2
miR-151a-5p	1.77	1.1	1.4	3.2
miR-199a-5p	2.54	1.1	1.9	4.2
miR-146a-5p	1.57	1.1	1.2	1.4
miR-29b-3p	(−1.35)	(−1.5)	(−1.7)	(−1.7)

Negative numbers (in parenthesis) indicated down-regulation of miRNA in amyotrophic lateral sclerosis relative to controls and positive numbers indicate up-regulation.

**Table 3:** Fold regulation of amyotrophic lateral sclerosis miRNA fingerprint, as determined by qPCR from L1CAM-enriched EV extractions of blood plasma

		Amyotrophic lateral sclerosis versus controls				
	*P*-value	*Z*-statistic	Median amyotrophic lateral sclerosis (*n* = 119)	Median control (*n* = 150)	Fold regulation	Regulation
miR-10b-5p	*P* < 0.00001	10.39	0.15	1.27	(−7.38)	Down-regulated
miR-4454	*P* < 0.00001	9.97	0.38	0.95	(−2.55)	Down-regulated
miR-199a-3p	*P* < 0.00001	9.76	2.74	1.07	2.03	Up-regulated
miR-151a-3p	*P* < 0.00001	9.49	3.39	1.18	2.09	Up-regulated
miR-151a-5p	*P* < 0.00001	8.46	2.37	1.02	1.77	Up-regulated
miR-199a-5p	*P* < 0.00001	9.02	6.00	0.93	2.54	Up-regulated
miR-146a-5p	*P* < 0.00001	8.66	1.81	1.04	1.57	Up-regulated
miR-29b-3p	*P* < 0.00001	4.87	0.78	1.05	(−1.35)	Down-regulated

		Parkinson’s disease versus amyotrophic lateral sclerosis				
	*P*-value	*Z*-statistic	Median Parkinson’s disease (*n* = 20)	Median amyotrophic lateral sclerosis (*n* = 119)	Fold regulation	Regulation
miR-10b-5p	*P* < 0.05	2.35	0.36	0.15	4.21	Up-regulated
miR-4454	*P* < 0.001	3.58	0.72	0.38	1.64	Up-regulated
miR-199a-3p	NS	0.71	2.54	2.74	(−1.06)	Down-regulated
miR-151a-3p	NS	0.95	3.34	3.39	(−1.20)	Down-regulated
miR-151a-5p	*P* < 0.05	2.55	1.74	2.37	(−1.27)	Down-regulated
miR-199a-5p	NS	1.08	5.31	6.00	(−1.23)	Down-regulated
miR-146a-5p	*P* < 0.00001	4.11	2.37	1.81	1.46	Up-regulated
miR-29b-3p	*P* < 0.05	2.46	0.62	0.78	(−1.37)	Down-regulated

		Primary lateral sclerosis versus amyotrophic lateral sclerosis				
	*P*-value	*Z*-statistic	Median primary lateral sclerosis (*n* = 42)	Median amyotrophic lateral sclerosis (*n* = 119)	Fold regulation	Regulation
miR-10b-5p	*P* < 0.05	2.41	0.08	0.15	(−1.80)	Down-regulated
miR-4454	*P* < 0.05	2.32	0.27	0.38	(−1.54)	Down-regulated
miR-199a-3p	*P* < 0.001	3.32	2.13	2.74	(−1.32)	Down-regulated
miR-151a-3p	*P* < 0.00001	4.30	2.22	3.39	(−1.37)	Down-regulated
miR-151a-5p	*P* < 0.05	2.32	0.27	0.38	(−1.54)	Down-regulated
miR-199a-5p	*P* < 0.00001	6.67	1.79	6.00	(−3.24)	Down-regulated
miR-146a-5p	*P* < 0.001	3.63	1.50	1.81	(−1.29)	Down-regulated
miR-29b-3p	*P* < 0.01	2.98	1.12	0.78	1.36	Up-regulated

*Z*-statistic was from a two-tailed Mann–Whitney *post hoc* analysis. Median values are reported as gene fold expression (2^(−ΔΔCq)^). *Z*-statistic is reported as absolute values.

**Figure 2:**
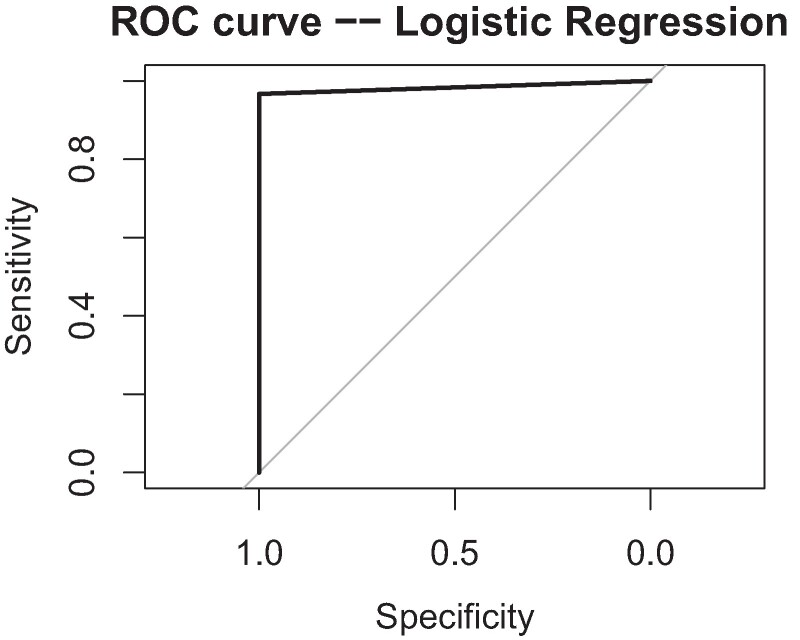
**Receiver operator characteristics (ROC) curve.** ROC curve calculated with a logistic regression model on 269 total observations with 119 amyotrophic lateral sclerosis and 150 control samples. Area under the curve (AUC) = 0.98.

Classification accuracy was tested using three orthogonal measures (Tables 4–6). Using only the current data of amyotrophic lateral sclerosis and healthy controls (*n* = 269) and a logistic regression model (Table 5) with a random data split, sensitivity and NPV were both at 100% while specificity and PPV were at 97% and 96%, respectively (Table 6). A ROC curve demonstrated an area under the curve (AUC) of 98% (Fig. 2). Deviance residuals for the model were as follows: min = −2.10, first quartile = −0.28, median = 0.03, third quartile = 0.24, max = 3.79, null deviance = 296.8 (215 degrees of freedom) and residual deviance = 96.3 (207 degrees of freedom). Akaike information criterion was 114.3, f1 score was 98% and the number of Fisher Scoring iterations was 7. A random forest analysis with a random split, also using only the current data, had good sensitivity, specificity, PPV and NPV all between 96 and 97% (Table 6). The best results in this analysis used only six of the eight miRNAs (mi-29b-3p and miR-146a-5p were removed), and the contribution of each remaining miRNA was determined (Table 4). When the same data were tested against a training data set pulled from an independent cohort of samples (Banack *et al*.^23^), sensitivity dropped to 82% but specificity remained at 97% (Table 6).

**Table 4:** Random forest machine learning analysis

	Mean decrease in Gini	
	Random split (*n* = 269)	Separate cohorts: train^23^ (*n* = 100); test (*n* = 269)
miR-10b-5p	33.6	9.0
miR-4454	21.4	9.6
miR-199a-3p	17.4	12.2
miR-151a-3p	15.8	4.9
miR-151a-5p	9.1	7.7
miR-199a-5p	7.8	6.0

Mean decrease in Gini is a measure of the contribution of each miRNA to the homogeneity of the random forest trees where an increased value represents a larger contribution to the classification criteria. Random split data are exclusively from the current data set with a 79% random split. Separate cohorts used the current data set to test against the training data, previously published,^23^ which used plasma from an independent sample cohort.

**Table 5:** Logistic regression model used to calculate receiver operator characteristics (ROC) curve

	Estimate Std.	Std. error	*Z* value	*Pr*(>|*z*|)
Intercept	−4.58	1.76	−2.61	0.009**
miR-10b-5p	−0.21	0.17	−1.26	0.21
miR-4454	−1.15	0.29	−3.98	6.84e-05***
miR-199a-3p	1.22	0.50	2.43	0.01*
miR-151a-3p	0.48	0.30	1.60	0.11
miR-151a-5p	1.17	0.37	3.19	0.001**
miR-199a-5p	−0.11	0.23	−0.47	0.64
miR-146a-5p	1.59	0.62	2.55	0.011*
miR-29b-3p	1.72	0.46	3.70	0.0002***

***P < 0.001, **P < 0.01, *P < 0.05.

**Table 6:** Three orthogonal models were used to interrogate amyotrophic lateral sclerosis classification between amyotrophic lateral sclerosis and healthy controls

	Sensitivity (%)	Specificity (%)	Positive predictive value (%)	Negative predictive value (%)	Overall accuracy (%)	# miRNA included	Sample size	Data source (details)
1. Random forest split	96	97	96	97	96	6	*n* = 269	Current data (79% split)
2. Random forest separate cohorts	82	97	95	87	90	6	*n* = 369	Current data (test, *n* = 269) + train data^23^ (*n* = 100)
3. Logistic regression	100	97	96	100	98	8	*n* = 269	Current data (80% split)

## Discussion

Amyotrophic lateral sclerosis patients and the neurologists that diagnose them need an amyotrophic lateral sclerosis diagnostic biomarker.^1^ It has been two centuries since Charles Bell first described the symptoms of amyotrophic lateral sclerosis^39^ and 150 years since Jean-Martin Charcot named the disease.^40^ Despite worldwide awareness of amyotrophic lateral sclerosis and a plethora of scientific research, patients are still faced with a significant diagnosis delay.^41^ The rapid progression of the disease increases the need for a quick and accurate diagnostic test from easily obtained biosamples. Adding a blood-based diagnostic test to confirm suspected amyotrophic lateral sclerosis would help neurologists and benefit patients. We have demonstrated the robust, reproducible, sensitive and accurate ability of an eight-miRNA fingerprint to diagnose amyotrophic lateral sclerosis and differentiate between amyotrophic lateral sclerosis and two other neurological diseases. We envision this biomarker as a quick secondary measure of disease diagnosis following a neurologist’s clinical evaluation. The enhanced diagnostic confidence provided by a biomarker would enable neurologists to diagnosis amyotrophic lateral sclerosis patients at an earlier stage of disease. This in turn would benefit patients by providing an opportunity for earlier use of disease modifying amyotrophic lateral sclerosis drugs.

In order to qualify as a new diagnostic biomarker with FDA approval, four criteria must be met: (i) a needs assessment; (ii) context of use; (iii) benefit/risk; and (iv) evidence to support qualification.^1,3^ The current data evaluate an eight-miRNA amyotrophic lateral sclerosis diagnostic fingerprint providing evidence for benefit/risk through classification algorithms with good discriminatory power. The high sensitivity, specificity, PPV and NPV suggest that the benefits of the biomarker in conjunction with clinical evaluation would outweigh any risk, particularly given the rapid rate of amyotrophic lateral sclerosis disease progression. Further evidence to support qualification includes consistent fold change calculations and highly different fold regulation between comparison groups as noted by a Mann–Whitney analysis (Table 3).

miRNAs represent good candidate biomarker targets due to their regulatory roles in essential cell functions. Many miRNAs have been identified as dysregulated in amyotrophic lateral sclerosis patients with ongoing research investigating their biomarker potential (e.g. Gomes *et al*.,^11^ J. Cheng *et al.*,^42^ Y.F. Cheng et al.,^43^ Hur *et al*.,^44^ Koike and Onodera,^45^ Liu *et al*.,^46^ Rizzuti *et al*.^47^ and Gama *et al*.^48^). Disparity between studies is a concern in miRNA research and can be attributed largely to tissues, methods, sample size and research focus with a resulting multiplicity of fine detail that can alter the end results between studies.^49,50^ Reproducibility in miRNA studies can be impacted by (i) pre-analytical variables including sample collection and processing procedures; (ii) RNA isolation, quantification and handling methods; (iii) lack of internal quality control measures; and (iv) data acquisition, processing and statistical analysis variability.^51^ Furthermore, few studies have been adequately validated using large samples sizes of unique patient cohorts. The nature of amyotrophic lateral sclerosis as a rare disease further complicates the ability of researchers to find access to large sample sizes which are necessary for the development of robust classification predictions.

We outline a method to enhance reproducible miRNA discovery intended for clinical applications. This robust amyotrophic lateral sclerosis biomarker was identified through a series of steps involving the extraction of neural-enriched extracellular vesicles, next-generation sequencing, qPCR verification, extensive internal quality control, multiple unique patient cohort testing and machine learning classification. This study represents the fourth experiment in a series^22,23^ using four unique patient cohorts all providing repeatable fold change and suggesting a robust, accurate and repeatable miRNA fingerprint for amyotrophic lateral sclerosis. The current cohort of samples represent blood plasma collected from several sources using generally accepted plasma collection protocols but without standardization. These samples, which are thus more heterogeneous in nature, varied in storage duration, amyotrophic lateral sclerosis stage, and diagnosing neurologist. This diversity in pre-analytical sample sources suggests that the miRNA fingerprint was not sensitive to specific blood plasma collection protocols, an assertion which is consistent with our prior study and increases the likelihood of future reproducibility.^23^

In comparison to all miRNA found to be dysregulated in amyotrophic lateral sclerosis, the eight miRNAs, found here to be valuable in combination, are a small subset of those identified. However, the eight-miRNA fingerprint has important links to amyotrophic lateral sclerosis biological pathways (Table 7). The generic miR-146a, without referring to the specific strand 146a-5p we identified, has been confirmed by others with similar up-regulation in muscle biopsies, brain and spinal cord^52,53^ but down-regulated in serum.^54^ miR-10b-5p did not significantly differ between amyotrophic lateral sclerosis and controls in muscle biopsies^55^ and was found increased in the brain and spinal cord,^53^ both differing from our findings in neural-enriched extracellular vesicles from blood plasma. We found that miR-29b-3b consistently down-regulated in amyotrophic lateral sclerosis plasma but miR-29b was found to be up-regulated in amyotrophic lateral sclerosis muscle biopsy tissues.^56^ miR-4454 has been found both in our studies and by others^57^ to be down-regulated in amyotrophic lateral sclerosis blood plasma; however, in serum, Lo *et al*.^58^ found miR-4454 to be up-regulated. miR-151a-5p was shown by others to be up-regulated in early amyotrophic lateral sclerosis but down-regulated with disease progression.^59,60^ miR-199a-3p was up-regulated in sporadic-amyotrophic lateral sclerosis blood plasma^43^ which is consistent with our results. Both miR-199a-3p and miR-199a-5p were shown to correlate with clinical amyotrophic lateral sclerosis parameters^60^ and further research may demonstrate prognostic value. Different tissue types and methods of miRNA detection largely explain the variation between research studies but other factors, as noted above, cannot be ruled out.

**Table 7:** Connection between miRNA biomarkers with amyotrophic lateral sclerosis biological processes and disease state

miRNA: current data + Banack *et al*.^22,23^	Biological process/pathways	Link to ALS and neurodegeneration
miR-151a-3p • Up-regulated	- Neuroprotective^61^	- Down-regulated in autism and schizophrenia^62,63^ - Dysregulated in PD^64^
miR-151a-5p • Up-regulated	- Oxidative stress^65^ - Cell viability^65^	- Up-regulated in early-stage ALS but down-regulated in end-stage ALS^59,60^ - Down-regulated in PD^66^
miR-146a-5p • Up-regulated	- Triggers motor neuron loss^67^ - Regulates neurofilament protein^68^ - Increases synaptic transmission^69^ - Reduces synaptic plasticity^70^ - Contributes to neuroinflammation^71-73^ - May function to alleviate neuropathic pain^74^ - Pre-miR-146a regulates mitochondria and inflammation^75^ - Impacts cellular bioenergetics^76^	- sALS: pathogenic, found in white blood cells, CSF and spinal cord^77^ - Up-regulated in ALS muscle biopsy^52^ - Up-regulated in ALS brain and spinal cord^53^ - Down-regulated in ALS serum^54^ - Down-regulated in AD plasma or serum^73,78-82^ - Up-regulated in AD brain, CSF^76,83-85^ - Up-regulated in multiple sclerosis CSF^86^
miR4454 • Down-regulated	- Predicted to impact neurogenesis, synapse formation and motor neuron integrity^58^	- Up-regulated in ALS serum^58^ - Specific to ALS, not dysregulated in PD^87^ - Found down-regulated in ALS blood plasma^57^
miR-10b-5p • Down-regulated	- Increases BDNF^88^ - Positive effect on memory and learning^89-91^ - Positive effect on synaptogenesis and neuron survival^89-91^ - Down-regulated in myoblasts proliferation and up-regulated in myoblasts differentiation^92^ - Down-regulation enhances HOXD10 and inactivates Rho/ROCK signalling pathway resulting in reduced neuronal apoptosis, inflammation and oxidative stress^93^	- Increased BDNF in ALS lymphocytes^89-91^ - Up-regulated in ALS brain and spinal cord^53^ - Dysregulated in PD^64^ - Up-regulated in HD^94^
miR-199a-3p • Up-regulated	- Negative effect on regeneration of damaged neurons^95^ - Decreases mTOR negatively affecting axon regeneration and plasticity^95,96^	- Correlated with clinical ALS parameters^60^ - Up-regulated in sALS plasma^43^ - Down-regulated in PD^94,97^
miR-199a-5p • Up-regulated	- Protective in spinal cord injury models^98-100^ - Promotes neurogenesis and neuronal differentiation^101^	- Correlated with clinical ALS parameters^60^ - Expression differentiates AD^60^
miR-29b-3p • Down-regulated	- Regulates pro-apoptotic/anti-apoptotic pathways^102^	- Up-regulated in ALS muscle biopsy^56^

ALS, amyotrophic lateral sclerosis; sALS, sporadic ALS; AD, Alzheimer’s disease; BDNF, brain-derived neurotrophic factor; CSF, cerebrospinal fluid; HOXD10, homeobox D10; mTOR, mammalian target of rapamycin; PD, Parkinson’s disease; Rho/ROCK, Rho-associated protein kinase.

The precise function of each miRNA and target pathways related to disease onset and progression are not yet clear. Prior research suggests that these eight miRNAs affect biological process consistent with neurodegenerative disease affecting such biological processes as oxidative stress, cell viability, motor neuron loss, synaptic transmission, neuron regeneration, neural inflammation and more (Table 7). However, many miRNAs have multiple targets^103^; including several miRNAs from different pathways can increase the robust nature and potential specificity of the test.^104^ Further research identifying the disease-related pathways involved is warranted.

Of particular research interest is finding blood-based biomarkers that can enhance amyotrophic lateral sclerosis clinical trials. Seven blood protein biomarkers are currently being used in ongoing amyotrophic lateral sclerosis trials,^14^ but only one, high-sensitivity cardiac troponin T (hs-cTnT),^105^ has promising potential as a diagnostic. It has not been shown to be as accurate as the current biomarker with the ability to distinguish amyotrophic lateral sclerosis from amyotrophic lateral sclerosis mimics noted as 0.70 AUC [95% confidence interval (CI) = 0.61–0.79] and its ability to differentiate amyotrophic lateral sclerosis from healthy controls as 0.88 AUC (95% CI = 0.70–0.97).^105^ The majority of research to date on amyotrophic lateral sclerosis diagnostics has been on neurofilament light chain which was initially considered as a diagnostic.^106-112^ However, since neurofilament light chain concentrations have been found to increase in blood plasma associated with many neurodegenerative diseases, it is perhaps better suited as a surrogate end-point in clinical trials. This is the current context in which neurofilaments are being employed.^12,108,111-114^ We have demonstrated the utility of this eight-miRNA fingerprint for amyotrophic lateral sclerosis diagnosis; however, it is not yet known if it can be informative concerning progression. The paucity of prognostic data allowing us to address this issue is a limitation of the research to date. The primary lateral sclerosis natural history study^26^ continues to provide additional samples and disease confirmation of probable cases leading to valuable prognostic data to support future research. We found highly significant differences in the miRNA expression between amyotrophic lateral sclerosis, Parkinson’s disease and primary lateral sclerosis (Table 3). However, small sample sizes limit our ability to accurately assess sensitivity and specificity, which require larger sample sizes.^38^ Small sample sizes of comparative neurological groups and the availability of additional amyotrophic lateral sclerosis-mimic samples are another limitation of this study.

## Conclusion

The eight-miRNA fingerprint described herein provides evidence of a strong relationship between this diagnostic biomarker and the gold standard method of clinical disease diagnosis. The analytical performance is good in three separate classification analyses (Table 6; sensitivity = 82, 96, 100; specificity = 97, 97, 97%) with important predictive value (PPV = 95–96%; NPV = 87–100%). The use of independent cohort groups in the machine learning algorithm, with one cohort used for training and another cohort for testing, provides support for the predictive value of the test. The significant differences found in the fold regulation between amyotrophic lateral sclerosis, Parkinson’s disease and primary lateral sclerosis further support the utility of this biomarker, but further work here is warranted to increase sample sizes. Four independent experiments have tested the eight-miRNA fingerprint to compare amyotrophic lateral sclerosis and non-neurological controls samples which used different patient samples, for a total sample size of 471.^22,23^ The analytical validation of the miRNA fingerprint in this study and prior research establishes that the biomarker test is both accurate and reproducible on blood plasma collected using multiple collection protocols. It is not sensitive to variation in standard blood plasma collection, storage or processing conditions which increases its potential for clinical diagnostic evaluation.^22-24,27^ Further validation of this biomarker using samples from prospective and longitudinal studies, as well additional neurological controls and amyotrophic lateral sclerosis-mimic diseases, would aid its future use and accelerate its introduction into the clinic and clinical trials.

## Supplementary Material

fcae268_Supplementary_Data

## Data Availability

The data that support the findings of this study are available from the corresponding author, P.C., upon reasonable request.
