# Supplementary material for: A microRNA diagnostic biomarker for amyotrophic lateral sclerosis
Source: Brain Commun. 2024 Sep 13;6(5):fcae268. doi: 10.1093/braincomms/fcae268 (PMC11398878; doi:10.1093/braincomms/fcae268)
Supplement: fcae268_Supplementary_Data [file fcae268_supplementary_data.pdf]

Supplementary Materials

Supplementary Table 1: cDNA synthesis conditions

Qiagen miRCURY LNA RT Kit

Cat. No. / ID: 339340

Step	Time	Temp (°C)
reverse transcription step	60 min	42
inactivation of reaction	5 min	95
Hold	∞	4

Supplementary Table 2: Qiagen GeneGlobe IDs for primers.

		Qiagen miRCURY LNA miRNA PCR Assays catalogue number: 339306	
		Name	GeneGlobe ID
	Targets & reference genes	hsa-miR-10b-5p	YP00205637
		hsa-miR-4454	YP02114119
		hsa-miR-199a-3p	YP00204536
		hsa-miR-151a-3p	YP00204576
		hsa-miR-151a-5p	YP00204007
		hsa-miR-199a-5p	YP00204494
		hsa-miR-146a-5p	YP00204688
		hsa-miR-29b-3p	YP00204679
		hsa-miR-126-5p	YP00206010
Spike-ins	RNA extraction efficiency	UniSp2	YP00203950
		UniSp4	YP00203953
		UniSp5	YP00203955
	Reverse transcription efficiency	UniSp6	YP00203954
		cel-miR-39-3p	YP00203952
	Sample Signal	hsa-miR-142-3p	YP00204291
		hsa-miR-451a	YP02119305
		hsa-miR-23a-3p	YP00204772
		hsa-miR-30c-5p	YP00204783
		hsa-miR-103a-3p	YP00204063
		hsa-miR-191-5p	YP00204306

Supplementary Table 3: Real-time qPCR reaction conditions

miRCURY LNA miRNA PCR Assay

Cat. No. / ID: 339306

Step	Time	Temp (°C)
PCR initial activation	2 min	95
2-step cycling		
Denaturation	10 secs	95
Combined annealing/extension	60 secs	56
Number of cycles	40	
Melt curve analysis increment 0.5°C		60-95

Supplementary Table 4: Haemolysis calculations, January 2023.

ΔCq > 7 indicates haemolysis may have occurred in the sample.

Eight samples returned ΔCq values > 7 but not so great to be excluded from downstream analysis.

Sample	C_q 23a-3p	Sample	C_q 451a	ΔC_q
S1	**31.39**	S1	**27.89**	✓ **3.50**
S2	**33.18**	S2	**29.05**	✓ **4.13**
S3	**33.00**	S3	**28.32**	✓ **4.68**
S4	**32.57**	S4	**27.50**	✓ **5.07**
S5	**32.34**	S5	**29.55**	✓ **2.79**
S6	**30.39**	S6	**25.00**	✓ **5.39**
S7	**27.77**	S7	**24.71**	✓ **3.06**
S8	**28.15**	S8	**23.74**	✓ **4.41**
S9	**34.46**	S9	**31.65**	✓ **2.81**
S10	**34.60**	S10	**31.00**	✓ **3.60**
S11	**31.60**	S11	**28.46**	✓ **3.14**
S12	**34.42**	S12	**31.77**	✓ **2.65**
S13	**28.83**	S13	**28.48**	✓ **0.35**
S14	**32.10**	S14	**27.28**	✓ **4.82**
S15	**31.73**	S15	**27.94**	✓ **3.79**
S16	**32.22**	S16	**28.54**	✓ **3.68**
S17	**32.13**	S17	**26.29**	✓ **5.84**
S18	**31.39**	S18	**26.06**	✓ **5.33**
S19	**32.10**	S19	**27.09**	✓ **5.01**
S20	**31.52**	S20	**28.46**	✓ **3.06**
S21	**31.09**	S21	**28.19**	✓ **2.90**
S22	**32.23**	S22	**28.48**	✓ **3.75**
S23	**29.62**	S23	**27.79**	✓ **1.83**
S24	**31.31**	S24	**25.56**	✓ **5.75**
S25	**28.64**	S25	**23.88**	✓ **4.76**
S26	**26.66**	S26	**23.13**	✓ **3.53**
S27	**29.32**	S27	**24.94**	✓ **4.38**
S28	**28.94**	S28	**23.68**	✓ **5.26**
S29	**31.41**	S29	**24.97**	✓ **6.44**
S30	**27.50**	S30	**22.89**	✓ **4.61**
S31	**29.43**	S31	**24.54**	✓ **4.89**
S32	**30.12**	S32	**25.79**	✓ **4.33**
S33	**34.09**	S33	**32.34**	✓ **1.75**
S34	**31.62**	S34	**31.76**	! **-0.14**
S35	**32.74**	S35	**31.71**	✓ **1.03**

S36	**35.32**	S36	**31.10**	✓	**4.22**
S37	**27.98**	S37	**24.75**	✓	**3.23**
S38	**29.34**	S38	**25.02**	✓	**4.32**
S39	**29.64**	S39	**26.21**	✓	**3.43**
S40	**29.05**	S40	**24.27**	✓	**4.78**

S41	**28.49**	S41	**24.93**	✓	**3.56**
S42	**30.02**	S42	**24.61**	✓	**5.41**
S43	**26.22**	S43	**23.11**	✓	**3.11**
S44	**26.97**	S44	**23.13**	✓	**3.84**
S45	**28.12**	S45	**23.74**	✓	**4.38**
S46	**27.08**	S46	**24.18**	✓	**2.90**
S47	**28.23**	S47	**24.39**	✓	**3.84**
S48	**28.21**	S48	**26.27**	✓	**1.94**
S49	**28.42**	S49	**25.21**	✓	**3.21**
S50	**29.39**	S50	**25.82**	✓	**3.57**
S51	**28.77**	S51	**26.37**	✓	**2.40**
S52	**26.32**	S52	**25.52**	✓	**0.80**
S53	**29.32**	S53	**27.01**	✓	**2.31**
S54	**28.79**	S54	**24.83**	✓	**3.96**
S55	**27.65**	S55	**23.68**	✓	**3.97**
S56	**25.14**	S56	**21.45**	✓	**3.69**
S57	**27.86**	S57	**24.10**	✓	**3.76**
S58	**27.01**	S58	**22.05**	✓	**4.96**
S59	**28.76**	S59	**25.29**	✓	**3.47**
S60	**27.98**	S60	**23.46**	✓	**4.52**
S61	**33.23**	S61	**31.33**	✓	**1.9**
S62	**32.67**	S62	**29.57**	✓	**3.1**
S63	**32.14**	S63	**29.18**	✓	**2.96**
S64	**32.35**	S64	**31.77**	✓	**0.58**
S65	**32.74**	S65	**29.64**	✓	**3.10**
S66	**33.12**	S66	**31.01**	✓	**2.11**
S67	**30.39**	S67	**27.32**	✓	**3.07**
S68	**31.00**	S68	**28.87**	✓	**2.13**
S69	**31.25**	S69	**30.57**	✓	**0.68**
S70	**31.54**	S70	**29.46**	✓	**2.08**
S71	**32.21**	S71	**30.67**	✓	**1.54**
S72	**31.99**	S72	**29.78**	✓	**2.21**
S73	**28.76**	S73	**26.63**	✓	**2.13**
S74	**29.14**	S74	**25.63**	✓	**3.51**

S75	29.58	S75	26.21	✔ 3.37
S76	28.72	S76	25.11	✔ 3.61
S77	29.87	S77	26.52	✔ 3.35
S78	33.35	S78	30.97	✔ 2.38
S79	34.25	S79	31.43	✔ 2.82
S80	28.27	S80	24.80	✔ 3.47
S81	28.10	S81	24.74	✔ 3.36
S82	28.40	S82	24.92	✔ 3.48
S83	28.72	S83	24.99	✔ 3.73
S84	28.14	S84	25.06	✔ 3.08
S85	28.74	S85	21.22	! 7.52

S86	28.11	S86	24.13	✔ 3.98
S87	28.02	S87	23.28	✔ 4.74
S88	29.13	S88	24.30	✔ 4.83
S89	29.40	S89	25.48	✔ 3.92
S90	27.91	S90	24.19	✔ 3.72
S91	31.22	S91	29.46	✔ 1.76
S92	32.02	S92	30.35	✔ 1.67
S93	31.29	S93	28.75	✔ 2.54
S94	33.51	S94	31.57	✔ 1.94
S95	31.01	S95	29.15	✔ 1.86
S96	33.05	S96	31.66	✔ 1.39
S97	31.84	S97	29.17	✔ 2.67
S98	31.40	S98	28.58	✔ 2.82
S99	33.21	S99	32.27	✔ 0.94
S100	33.29	S100	29.39	✔ 3.90
S101	31.92	S101	30.18	✔ 1.74
S102	29.53	S102	28.78	✔ 0.75
S103	29.32	S103	26.08	✔ 3.24
S104	24.61	S104	27.02	! -2.41
S105	33.12	S105	29.66	✔ 3.46
S106	27.14	S106	22.71	✔ 4.43
S107	27.75	S107	20.29	! 7.46
S108	28.58	S108	25.11	✔ 3.47
S109	28.67	S109	23.74	✔ 4.93
S110	26.28	S110	23.10	✔ 3.18
S111	31.28	S111	27.15	✔ 4.13
S112	28.41	S112	24.36	✔ 4.05
S113	28.38	S113	26.07	✔ 2.31

S114	**28.53**	S114	**22.24**	✔	**6.29**
S115	**28.31**	S115	**22.18**	✔	**6.13**
S116	**29.27**	S116	**24.27**	✔	**5.00**
S117	**29.20**	S117	**23.90**	✔	**5.30**
S118	**27.95**	S118	**25.21**	✔	**2.74**
S119	**28.93**	S119	**24.75**	✔	**4.18**
S120	**29.14**	S120	**26.18**	✔	**2.96**
S121	**30.46**	S121	**24.85**	✔	**5.61**
S122	**28.13**	S122	**24.07**	✔	**4.06**
S123	**34.52**	S123	**29.97**	✔	**4.55**
S124	**33.90**	S124	**31.94**	✔	**1.96**
S125	**32.59**	S125	**32.96**	!	**-0.37**
S126	**33.66**	S126	**30.75**	✔	**2.91**
S127	**29.65**	S127	**26.11**	✔	**3.54**
S128	**31.34**	S128	**23.74**	!	**7.60**
S129	**32.77**	S129	**30.68**	✔	**2.09**
S130	**33.84**	S130	**31.85**	✔	**1.99**

S131	**32.56**	S131	**30.73**	✔	**1.83**
S132	**33.91**	S132	**31.34**	✔	**2.57**
S133	**29.31**	S133	**24.09**	✔	**5.22**
S134	**28.02**	S134	**24.33**	✔	**3.69**
S135	**29.43**	S135	**24.25**	✔	**5.18**
S136	**30.80**	S136	**24.07**	✔	**6.73**
S137	**30.21**	S137	**24.14**	✔	**6.07**
S138	**30.01**	S138	**25.24**	✔	**4.77**
S139	**30.44**	S139	**26.70**	✔	**3.74**
S140	**29.44**	S140	**25.98**	✔	**3.46**
S141	**29.03**	S141	**20.37**	!	**8.66**
S142	**29.48**	S142	**25.56**	✔	**3.92**
S143	**32.71**	S143	**31.30**	✔	**1.41**
S144	**33.12**	S144	**31.94**	✔	**1.18**
S145	**33.14**	S145	**31.15**	✔	**1.99**
S146	**32.19**	S146	**29.47**	✔	**2.72**
S147	**31.52**	S147	**29.77**	✔	**1.75**
S148	**31.18**	S148	**28.89**	✔	**2.29**
S149	**31.69**	S149	**30.03**	✔	**1.66**
S150	**32.21**	S150	**29.65**	✔	**2.56**
S151	**26.86**	S151	**24.15**	✔	**2.71**
S152	**26.89**	S152	**23.93**	✔	**2.96**

S153	**26.49**	S153	**22.77**	✓	**3.72**
S154	**28.91**	S154	**24.53**	✓	**4.38**
S155	**26.88**	S155	**23.03**	✓	**3.85**
S156	**28.04**	S156	**20.25**	!	**7.79**
S157	**29.35**	S157	**21.84**	!	**7.51**
S158	**29.15**	S158	**23.53**	✓	**5.62**
S159	**29.02**	S159	**20.72**	!	**8.30**
S160	**27.92**	S160	**24.40**	✓	**3.52**
S161	**28.38**	S161	**24.72**	✓	**3.66**
S162	**27.42**	S162	**22.08**	✓	**5.34**
S163	**32.79**	S163	**31.18**	✓	**1.61**
S164	**32.32**	S164	**30.20**	✓	**2.12**
S165	**32.80**	S165	**30.02**	✓	**2.78**
S166	**32.66**	S166	**30.18**	✓	**2.48**
S167	**32.13**	S167	**30.22**	✓	**1.91**
S168	**31.57**	S168	**30.13**	✓	**1.44**
S169	**31.62**	S169	**28.31**	✓	**3.31**
S170	**32.26**	S170	**30.22**	✓	**2.04**
S171	**32.09**	S171	**29.88**	✓	**2.21**
S172	**32.32**	S172	**30.16**	✓	**2.16**
S173	**30.96**	S173	**27.06**	✓	**3.90**
S174	**32.46**	S174	**28.04**	✓	**4.42**
S175	**31.97**	S175	**29.18**	✓	**2.79**

S176	**32.70**	S176	**31.15**	✓	**1.55**
S177	**31.74**	S177	**29.96**	✓	**1.78**
S178	**31.39**	S178	**29.44**	✓	**1.95**
S179	**31.10**	S179	**27.93**	✓	**3.17**
S180	**32.26**	S180	**29.08**	✓	**3.18**
S181	**31.23**	S181	**29.24**	✓	**1.99**
S182	**31.48**	S182	**29.45**	✓	**2.03**
S183	**26.79**	S183	**23.59**	✓	**3.20**
S184	**28.32**	S184	**21.19**	!	**7.13**
S185	**26.98**	S185	**22.78**	✓	**4.20**
S186	**29.13**	S186	**23.10**	✓	**6.03**
S187	**28.33**	S187	**23.19**	✓	**5.14**
S188	**29.72**	S188	**25.04**	✓	**4.68**
S189	**27.07**	S189	**22.20**	✓	**4.87**
S190	**28.02**	S190	**22.85**	✓	**5.17**
S191	**30.68**	S191	**28.36**	✓	**2.32**

S192	**31.92**	S192	**31.02**	**0.90**
S193	**31.56**	S193	**28.91**	**2.65**
S194	**33.17**	S194	**32.11**	**1.06**
S195	**31.97**	S195	**30.25**	**1.72**
S196	**31.56**	S196	**31.16**	**0.40**
S197	**31.24**	S197	**28.54**	**2.70**
S198	**32.98**	S198	**32.21**	**0.77**
S199	**33.02**	S199	**32.31**	**0.71**
S200	**33.11**	S200	**30.33**	**2.78**
S201	**32.26**	S201	**31.33**	**0.93**
S202	**31.43**	S202	**28.99**	**2.44**
S203	**28.56**	S203	**24.21**	**4.35**
S204	**30.04**	S204	**24.07**	**5.97**
S205	**27.98**	S205	**23.51**	**4.47**
S206	**28.83**	S206	**24.34**	**4.49**
S207	**28.31**	S207	**23.25**	**5.06**
S208	**26.03**	S208	**22.03**	**4.00**
S209	**26.53**	S209	**24.62**	**1.91**
S210	**28.24**	S210	**23.11**	**5.13**
S211	**27.62**	S211	**25.22**	**2.40**
S212	**27.44**	S212	**23.34**	**4.10**
S213	**24.87**	S214	**24.03**	**0.84**
S214	**27.67**	S213	**23.90**	**3.77**
S215	**32.24**	S215	**28.85**	**3.39**
S216	**32.39**	S216	**30.15**	**2.24**
S217	**31.88**	S217	**29.63**	**2.25**
S218	**32.84**	S218	**29.20**	**3.64**
S219	**32.58**	S219	**30.31**	**2.27**
S220	**31.91**	S220	**30.74**	**1.17**

S221	**32.58**	S221	**30.14**	**2.44**
S222	**32.86**	S222	**28.79**	**4.07**

Supplementary Table 5: Haemolysis calculations, June/July 2023.

ΔCq > 7 indicates haemolysis may have occurred in the sample.

Five samples returned ΔCq values > 7 but not so great to be excluded from downstream analysis.

Sample	Plate	C_q 23a-3p	Sample	Plate	C_q 451a	ΔC_q	from same plate
1	9	31.75	1	9	29.41	2.34	Yes
1	14	30.62	1	14	29.09	1.53	Yes
2	9	33.5	2	9	32.03	1.47	Yes
3	9	32.95	3	9	31.41	1.54	Yes
4	9	32.79	4	9	31.29	1.5	Yes
4	14	31.77	4	14	30.57	1.2	Yes
5	9	32.63	5	9	31.11	1.52	Yes
6	8	32.79	6	8	32.02	0.77	Yes
6	14	31.91	6	14	30.93	0.98	Yes
6	16	32.59	6	16	31.23	1.36	Yes
7	8	32.03	7	8	29.53	2.5	Yes
7	16	31.55	7	16	28.9	2.65	Yes
8	9	33.55	8	9	32.09	1.46	Yes
9	9	29.58	9	9	25.64	3.94	Yes
10	16	27.18	10	16	19.82	7.36	Yes
10	8	27.45	10	8	19.36	8.09	Yes
11	9	28.39	11	9	21.33	7.06	Yes
12	9	28.04	12	9	22.42	5.62	Yes
13	16	26.96	13	16	24	2.96	Yes
14	16	27.11	14	16	21.75	5.36	Yes
15	16	26	15	16	19.9	6.1	Yes
15	8	26.19	15	8	20.46	5.73	Yes
16	16	30.08	16	16	23.51	6.57	Yes
17	16	28.68	17	16	21.44	7.24	Yes
18	16	25.69	18	16	21.4	4.29	Yes
19	16	27.24	19	16	24.28	2.96	Yes
20	16	29.47	20	16	25.5	3.97	Yes
21	9	28.17	21	9	24.29	3.88	Yes
22	8	29.82	22	8	23.69	6.13	Yes
23	9	28.67	23	9	22.22	6.45	Yes
24	9	27.92	24	9	23.8	4.12	Yes
25	10	25.87	25	10	21.78	4.09	Yes
26	10	26.95	26	10	20.35	6.6	Yes
27	10	26.76	27	10	21.5	5.26	Yes
28	10	26.65	28	10	22.01	4.64	Yes
29	8	26.96	29	8	24.43	2.53	Yes
29	10	26.83	29	10	23.35	3.48	Yes
30	10	26.82	30	10	25.32	1.5	Yes
31	10	27.28	31	10	22.08	5.2	Yes
32	10	28.24	32	10	21.34	6.9	Yes
51	10	30.92	51	10	24.9	6.02	Yes
51	14	29.98	51	14	24.37	5.61	Yes
52	10	30.25	52	10	23.07	7.18	Yes
53	10	27.55	53	10	22.12	5.43	Yes
54	10	27.97	54	10	24.84	3.13	Yes
55	10	27.85	55	10	25.22	2.63	Yes
56	10	30.71	56	10	27.96	2.75	Yes
56	10	28.66	56	10	24.63	4.03	Yes
64	10	29.99	64	10	28.23	1.76	Yes
64	15	30.55	64	15	27.75	2.8	Yes
66	10	26.02	66	10	23.85	2.17	Yes
66	15	30.23	66	15	28.2	2.03	Yes
69	8	26.26	69	8	24.45	1.81	Yes
69	10	26.07	69	10	22.17	3.9	Yes
69	15	26.08	69	15	23.53	2.55	Yes
70	7	26.65	70	7	22.54	4.11	Yes
70	15	26.07	70	15	21.99	4.08	Yes
71	10	27.83	71	10	22.67	5.16	Yes
71	14	27.22	71	14	22.16	5.06	Yes
72	10	29.85	72	10	23.35	6.5	Yes
72	15	27.93	72	15	23.19	4.74	Yes
73	10	28.23	73	10	25.44	2.79	Yes
73	15	28.43	73	15	25.68	2.75	Yes
75	10	31.85	75	10	27.3	4.55	Yes
75	15	31.41	75	15	27.25	4.16	Yes
76	10	31.04	76	10	28.2	2.84	Yes
76	15	31.31	76	15	28.13	3.18	Yes
77	10	29.74	77	10	28.65	1.09	Yes
77	15	29.44	77	15	28.67	0.77	Yes
79	8	30.81	79	8	28.56	2.25	Yes
79	11	30.86	79	11	28.13	2.73	Yes
80	11	29.84	80	11	26.68	3.16	Yes
81	11	27.79	81	11	24.96	2.83	Yes
82	8	26.25	82	8	24.3	1.95	Yes
82	11	26.14	82	11	23.15	2.99	Yes
83	11	32.12	83	11	29.24	2.88	Yes
84	11	31.42	84	11	27.12	4.3	Yes
85	11	31.29	85	11	27.06	4.23	Yes
86	11	31.01	86	11	28.06	2.95	Yes
87	11	31.53	87	11	28.15	3.38	Yes
88	8	30.86	88	8	28.1	2.76	Yes
88	11	31.64	88	11	27.07	4.57	Yes
89	11	31.32	89	11	26.18	5.14	Yes
90	11	31.14	90	11	26.23	4.91	Yes
91	11	30.68	91	11	26.90	3.78	Yes
92	8	27.53	92	8	27.87	-0.34	Yes
92	11	27.26	92	11	26.73	0.53	Yes
93	8	32.17	93	8	29.25	2.92	Yes
93	11	31.6	93	11	28.14	3.46	Yes
94	11	31.29	94	11	28.06	3.23	Yes
95	11	31.26	95	11	29.55	1.71	Yes
96	11	33.66	96	11	28.80	4.86	Yes
97	11	26.94	97	11	24.36	2.58	Yes
98	11	28.16	98	11	25.90	2.26	Yes
99	11	27.65	99	11	24.88	2.77	Yes
100	11	29.7	100	11	24.67	5.03	Yes
101	11	29.36	101	11	24.29	5.07	Yes
102	11	29.06	102	11	23.52	5.54	Yes
103	11	28.71	103	11	24.46	4.25	Yes
104	11	30.53	104	11	24.17	6.36	Yes
105	11	26.04	105	11	19.11	6.93	Yes
106	11	26.26	106	11	24.33	1.93	Yes
107	13	26.47	107	13	23.61	2.86	Yes
108	13	25.78	108	13	23.81	1.97	Yes
109	13	27.62	109	13	21.00	6.62	Yes
110	13	25.49	110	13	20.32	5.17	Yes
111	13	26.35	111	13	22.64	3.71	Yes
112	13	26.05	112	13	24.35	1.7	Yes
113	13	29.11	113	13	24.54	4.57	Yes
114	13	32.02	114	13	29.19	2.83	Yes
115	13	27.21	115	13	24.80	2.41	Yes
116	13	29.57	116	13	23.58	5.99	Yes
117	13	29.50	117	13	25.56	3.94	Yes
118	13	29.70	118	13	22.49	7.21	Yes
119	13	28.82	119	13	24.93	3.89	Yes
120	13	28.04	120	13	24.84	3.2	Yes
121	13	25.24	121	13	23.39	1.85	Yes
122	12	26.83	122	12	22.54	4.29	Yes
123	12	26.31	123	12	21.03	5.28	Yes
124	12	24.58	124	12	19.33	5.25	Yes
125	12	26.01	125	12	21.62	4.39	Yes
126	12	28.38	126	12	24.65	3.73	Yes
127	12	27.05	127	12	21.60	5.45	Yes
128	12	25.55	128	12	22.74	2.81	Yes
129	12	26.65	129	12	23.18	3.47	Yes
130	12	26.00	130	12	21.34	4.66	Yes
131	12	31.39	131	12	29.37	2.02	Yes
132	12	28.99	132	12	24.45	4.54	Yes
133	12	30.43	133	12	26.76	3.67	Yes
134	12	31.29	134	12	28.43	2.86	Yes
135	12	31.02	135	12	29.35	1.67	Yes
136	12	27.35	136	12	24.14	3.21	Yes
137	12	27.14	137	12	24.79	2.35	Yes
138	12	26.20	138	12	24.14	2.06	Yes
139	12	30.72	139	12	27.00	3.72	Yes
140	12	30.73	140	12	26.42	4.31	Yes
141	12	31.44	141	12	28.69	2.75	Yes
142	12	31.76	142	12	27.15	4.61	Yes
142	14	31.1	142	14	26.9	4.2	Yes
143	12	31.03	143	12	28.32	2.71	Yes
144	12	31.22	144	12	28.15	3.07	Yes
145	12	30.75	145	12	26.50	4.25	Yes
146	13	32.50	146	13	29.47	3.03	Yes
147	9	29.04	147	9	24.61	4.43	Yes
147	13	30.28	147	13	25.23	5.05	Yes
148	9	28.28	148	9	22.76	5.52	Yes
148	13	28.57	148	13	23.02	5.55	Yes
149	9	27.67	149	9	25.01	2.66	Yes
149	13	28.00	149	13	25.11	2.89	Yes
150	9	28.09	150	9	22.41	5.68	Yes
150	13	27.64	150	13	22.27	5.37	Yes
151	13	27.42	151	13	24.83	2.59	Yes
152	13	28.27	152	13	24.62	3.65	Yes
153	13	29.18	153	13	24.02	5.16	Yes
154	13	26.40	154	13	25.54	0.86	Yes
155	13	28.04	155	13	24.07	3.97	Yes
156	13	27.78	156	13	24.50	3.28	Yes
157	13	27.99	157	13	22.97	5.02	Yes
158	13	26.12	158	13	21.25	4.87	Yes
159	13	26.01	159	13	21.49	4.52	Yes
160	13	26.36	160	13	24.45	1.91	Yes
161	14	32.22	161	14	32.6	-0.38	Yes
162	14	30.71	162	14	29.01	1.7	Yes
163	14	30.02	163	14	28.86	1.16	Yes
164	14	29.67	164	14	25.23	4.44	Yes
165	14	32.45	165	14	31.11	1.34	Yes
166	14	31.07	166	14	26.86	4.21	Yes
167	14	29.59	167	14	24.76	4.83	Yes
168	14	30.14	168	14	27.59	2.55	Yes
169	14	28.1	169	14	22.68	5.42	Yes
170	14	29.55	170	14	24.59	4.96	Yes
171	14	26.74	171	14	24.91	1.83	Yes
172	14	27.15	172	14	25.3	1.85	Yes
